# Loop-Structured PEG-Lipoconjugate Enhances siRNA Delivery Mediated by Liner-PEG Containing Liposomes

**DOI:** 10.3390/molecules30204127

**Published:** 2025-10-19

**Authors:** Daniil V. Gladkikh, Elena V. Shmendel, Darya M. Makarova, Mikhail A. Maslov, Marina A. Zenkova, Elena L. Chernolovskaya

**Affiliations:** 1 Institute of Chemical Biology and Fundamental Medicine SB RAS, Lavrentieva Ave. 8, 630090 Novosibirsk, Russia; 2 Lomonosov Institute of Fine Chemical Technologies, MIREA—Russian Technological University, Vernadsky Ave. 86, 119571 Moscow, Russiamamaslov@mail.ru (M.A.M.)

**Keywords:** siRNA delivery, liposomes, folate lipoconjugate, PEG lipoconjugate, intracellular accumulation, circulation in the blood stream

## Abstract

Therapeutics involving small interfering RNA (siRNA) have enormous potential for treating a number of diseases, but their effective delivery to target cells remains a major challenge. We studied the influence of the structure and combination of targeted (folate conjugated, F13) and shield lipoconjugates (P1500, diP1500) on the ability of cationic liposomal formulations based on the 2X3-DOPE system to deliver siRNA into cells in vitro and in vivo. The loop-structured PEG lipoconjugate equipped with two hydrophobic anchor groups (diP1500) demonstrated superior performance across multiple evaluation criteria. The F13/diP1500 composition maintained a compact particle size (126.0 ± 23.0 nm), while F13/P1500 with the same PEG chain equipped with one anchor group maintained an increased particle size of 241.8 ± 65.7 nm. Most critically, F13/diP1500 preserved substantial positive surface charges (21.6–30.5 mV) across all N/P ratios, demonstrating superior ability in avoid the “PEG dilemma”, whereas F13/P1500 suffered substantial charge neutralization (3.9–9.1 mV). Competitive inhibition with free folate confirmed receptor-mediated cellular accumulation of siRNA mediated by F13 containing liposomal compositions. In vivo biodistribution revealed statistically significant circulation advantages: DSPE-PEG2000/diP1500 achieved the highest plasma concentration at 15 min (1.84 ± 0.01 pmol/mL), representing the first direct in vivo comparison of compositions with PEG lipoconjugates of the same length, but formed different structures in the liposomes due to the presence of one or two anchor groups. Our findings provide critical insights for the rational design of targeted liposomal delivery systems, highlighting the importance of balanced optimization between folate targeting functionality and PEG shielding for effective siRNA delivery both in vitro and in vivo.

## 1. Introduction

The development of effective delivery systems for therapeutic nucleic acids represents one of the most pressing challenges in modern pharmacology and biomedicine [1]. Small interfering RNAs (siRNAs) have established themselves as promising therapeutic agents for selective gene expression silencing, as evidenced by the FDA approval of six siRNA-based drugs: patisiran (2018), givosiran (2019), lumasiran (2020), inclisiran (2021), vutrisiran (2022), and nedosiran (2023) [2,3,4]. Despite significant progress in RNAi therapeutics, the main challenge remains the development of safe and effective delivery systems that ensure the targeted transport of nucleic acids to target cells [5].

The landscape of nucleic acid delivery encompasses diverse technological platforms, each with distinct advantages and limitations. Lipid nanoparticles (LNPs) based on ionizable lipids have demonstrated remarkable clinical success, exemplified by mRNA COVID-19 vaccines [6,7]. However, LNP systems typically lack active targeting capabilities and rely on passive accumulation via the enhanced permeability and retention (EPR) effect, which has been proven to be less reliable in solid tumors than initially hoped. Polymeric delivery systems, including polyethylenimine (PEI) and PLGA-based carriers, offer advantages in controlled release and chemical versatility but face challenges with toxicity and immunogenicity [8,9,10]. Inorganic nanocarriers such as mesoporous silica and selenium nanoparticles provide unique properties useful for theranostic applications [11,12], however, concerns regarding long-term biodegradability and clinical translatability persist. Viral vectors offer high transfection efficiency but pose risks of immunogenicity and insertional mutagenesis [13]. Hybrid nanostructures combining lipid and polymer components represent an emerging approach to integrate advantages of different platforms. These sophisticated systems demonstrate the field’s movement toward multifunctional nanocarriers that address multiple delivery barriers simultaneously, aligning with the concept of nanotheranostics, where diagnostic and therapeutic capabilities are unified [14,15].

Cationic liposomes represent one of the most promising platforms for siRNA delivery due to their ability to efficiently form complexes with negatively charged nucleic acids, favorable safety profile, and potential for modification to improve the pharmacokinetic properties [16,17]. Special attention has been paid to systems based on the polycationic amphiphile 2X3 (1,26-bis(cholest-5-en-3β-yloxycarbonylamino)-7,11,16,20-tetraazahexacosan tetrahydrochloride) in combination with the helper lipid dioleoylphosphatidylethanolamine (DOPE), which demonstrate high efficiency in delivering various types of nucleic acids both in vitro and in vivo [18,19].

Within this diverse landscape of delivery platforms, cationic liposome-based systems occupy a distinctive niche, offering a combination of efficient nucleic acid complexation, biocompatibility, ease of manufacturing, and amenability to active targeting modification. The 2X3/DOPE platform utilized in this study represents a cholesterol-based cationic liposome system that differs fundamentally from ionizable lipid-based LNPs: while LNPs rely on pH-dependent ionization for endosomal escape, our system employs DOPE-mediated membrane fusion, and unlike passive LNP accumulation, our approach incorporates folate receptor-mediated active targeting.

One of the key approaches for optimizing liposomal delivery systems is modification of the liposome surface with polyethylene glycol (PEG), which allows for increased circulation time in the bloodstream and improved biodistribution through the enhanced permeability and retention (EPR) effect [20,21]. However, PEGylation can have a dual impact on delivery efficiency: on the one hand, it reduces the transfection activity of liposomes in vitro and impairs the release of therapeutic cargo from endosomes; on the other hand, it improves the pharmacokinetic characteristics and reduces toxicity [22,23]. This inherent trade-off—known as the “PEG dilemma”—represents a fundamental challenge that transcends platform differences, affecting LNPs, polymeric systems, and liposomes equally [24]. Solutions to the PEG dilemma through architectural innovations are therefore broadly applicable across delivery platforms and represent a critical area for advancing nanomedicine.

To enhance the specificity of siRNA delivery to tumor cells, the use of folate-containing lipoconjugates is of particular interest [25]. Folate receptors demonstrate overexpression on the surface of various epithelial tumor cells including tumors of the ovary, uterus, lung, kidney, breast, colon, prostate, and brain [26,27]. Compared with other targeting ligands, folic acid offers several advantages: low immunogenicity, rapid tumor penetration, high affinity to a wide range of tumors, chemical stability, and ease of production [28].

We have previously developed an effective nucleic acid delivery system based on 2X3-DOPE, hereafter 2X3, liposomes modified with folate-containing lipoconjugates, which provides targeted delivery to tumors with high levels of folate receptor expression [29,30]. It has been demonstrated that folate-equipped cationic liposomes significantly enhance the delivery efficiency of anti-MDR1 siRNA to tumors and increase the effectiveness of chemotherapy in vivo [31]. The previously studied liposomes contained a lipoconjugate in which folate was attached to a PEG chain with a molecular weight of 800 Da. As a result, the addition of shielding lipoconjugates with PEG of similar or greater length could prevent the interaction of folate with the receptor on the cell surface, limiting the potential for optimizing both circulation and targeting simultaneously.

The aim of the present study was to comprehensively investigate the influence of cationic liposome composition on the efficiency of siRNA delivery in vitro and in vivo. This work examined the effect of the PEG-lipoconjugate structure on the physicochemical properties of liposomes equipped with the folate-PEG2000 lipoconjugate, evaluating their transfection efficiency and circulation dynamics in the bloodstream. Particular attention was paid to the comparative analysis of the effectiveness of various liposomes. By demonstrating superior performance of cyclic PEG structures over linear counterparts of identical molecular weight, this work provides insights relevant to the broader field of PEGylated nanomedicine and addresses a universal challenge that limits the efficacy of targeted nanocarriers across multiple technological platforms. The architectural innovation of loop-structured PEG modifications could, in principle, be adapted to various nanocarrier systems, potentially informing the design of next-generation delivery systems for diverse therapeutic applications.

## 2. Results and Discussion

### 2.1. Characteristics of the Liposomes and Their Complexes with siRNA

The study utilized six distinct liposomal formulations based on 2X3/DOPE—a cholesterol-derived cationic lipid core system (Table 1). 2X3/DOPE, composed of the polycationic lipid 1,26-bis(cholest-5-en-3β-yloxycarbonylamino)-7,11,16,20-tetraazahexacosan tetrahydrochloride (2X3) combined with helper lipid 1,2-dioleoyl-sn-glycero-3-phosphoethanolamine (DOPE) at a 1:2 molar ratio, served as the reference formulation (here and after 2X3). This cholesterol-based cationic amphiphile contains four positively charged amino groups, enabling electrostatic complexation with negatively charged siRNA phosphate groups.

**Figure 1:**
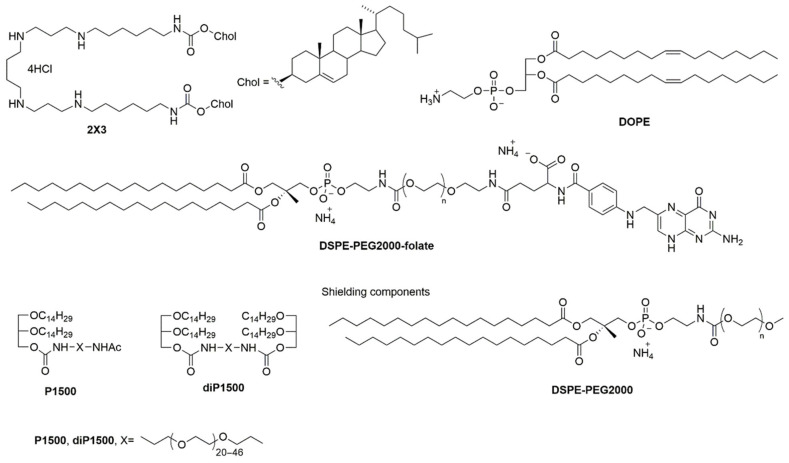
Structures of the liposomal components.

All targeted formulations studied included DSPE-PEG2000-folate (2 mol%) for implementation of receptor-mediated targeting: F13 liposomes contained only the core system and the folate conjugate (Figure 1), while the F13/P1500 and F13/diP1500 liposomes contained the additional shielding components P1500 or diP1500 (2 mol%) equipped with PEG with a molecular mass of 1500 Da. The length of the PEG chain in the shield components was chosen to prevent them from interfering with the interaction of folate attached to a longer linker with a folate receptor on the cell surface. The key distinction between P1500 and diP1500 lies in their architecture: P1500 features linear PEG-1500 chains attached to a 1,2-di-*O*-ditetradecyl-rac-glycerol anchor, while diP1500 employs loop-like PEG-1500 with 1,2-di-*O*-ditetradecyl-*rac*-glycerol anchor groups located at both chain termini (Figure 1).

In the control folate-free DSPE-PEG2000/P1500 and DSPE-PEG2000/diP1500 formulations, folate lipoconjugate was replaced with conventional DSPE-PEG2000 (2 mol%) while maintaining identical P1500 or diP1500 shielding components. This design enabled the direct assessment of folate targeting versus PEGylation effects while maintaining comparable surface architectures across formulations.

Determination of the physicochemical characteristics of the obtained liposomes and their complexes with siRNA showed that the basic folate-targeted F13 liposomes demonstrated (Table 1) moderate particle size (128.6 ± 5.9 nm), consistent with the optimal parameters for systemic delivery, as particles between 100 and 200 nm exhibit favorable pharmacokinetic profiles while avoiding rapid renal clearance [16]. At the same time, F13 is characterized by high polydispersity (0.7), indicating substantial heterogeneity in the particle population, since liposomal formulations with polydispersity values above 0.3 are typically considered heterogeneous [32]. The zeta potential of 32.4 ± 9.0 mV provides an adequate positive surface charge for nucleic acid complexation, though the large standard deviation indicates variability in the surface charge distribution across the particle population.

The incorporation of the linear P1500 lipoconjugate (F13/P1500) resulted in an increase in empty liposome size to 241.8 ± 65.7 nm, accompanied by enhanced surface charge (39.2 ± 8.1 mV). This size increase reflects the formation of an extended PEG corona and potential inter-particle aggregation mediated by PEG chain interactions, which is consistent with the literature data on the influence of structural modifications of cationic lipids on particle assembly dynamics [21,33]. The improvement in monodispersity aligns with reports on the stabilizing effect of PEG modification [34].

In contrast, the loop-structured diPEG1500 modification (F13/diP1500) maintained a more compact particle size (126.0 ± 23.0 nm) with comparable surface charge (28.8 ± 12.8 mV), suggesting more efficient surface organization of the loop PEG architecture. This represents a novel contribution to the field, as most studies have focused on linear PEG modifications. The superior size control achieved with loop-structured PEG may result from reduced steric hindrance and more efficient packing at the liposome surface.

The folate-free control liposomes demonstrated distinct size profiles. DSPE-PEG2000/P1500 showed a compact size (116.1 ± 17.9 nm) with moderate surface charge (26.8 ± 4.7 mV), while DSPE-PEG2000/diP1500 exhibited an increased size and variability (198.0 ± 99.2 nm) despite similar surface properties. The reference 2X3 system maintained the smallest particle size (88.0 ± 7.1 nm) and optimal surface charge (39.5 ± 2.2 mV), confirming its efficient self-assembly properties, which is consistent with our previous reports [29,35].

The complexation with siRNA at different N/P ratios revealed structure-specific assembly patterns. F13 liposomes showed relatively stable lipoplex sizes across N/P ratios (144.6–158.2 nm) with minimal size variation and consistent surface charge reduction upon siRNA binding. The zeta potential decreased from 32.4 mV to the 23.1–35.4 mV range, indicating efficient charge neutralization while maintaining positive surface characteristics necessary for cellular interactions. This charge reduction pattern is consistent with established principles of electrostatic complexation between cationic lipids and anionic siRNA [32,36], as the optimal surface charge balance of cationic lipoplexes is critical for cellular uptake while avoiding excessive cytotoxicity [37].

F13/P1500 complexes demonstrated pronounced N/P ratio dependence with dramatic zeta potential reduction. At the 4/1 ratio, the surface charge dropped to 3.9 ± 3.3 mV, suggesting near-complete charge neutralization that may compromise the cellular uptake efficiency. Progressive size reduction with increasing N/P ratio (from 201.5 nm at 4/1 to 171.7 nm at 8/1) indicates more compact lipoplex formation at higher lipid concentrations. The consistently low surface charges across all ratios (3.9–9.1 mV) represent a significant limitation for this formulation and align with studies demonstrating that excessive PEG density can shield positive charges necessary for cellular interaction—a phenomenon known as the “PEG dilemma” [23].

The F13/diP1500 system exhibited more favorable complexation behavior with maintained positive surface charges (21.6–30.5 mV) across all N/P ratios. Complex sizes showed ratio-dependent variation (138.9–214.7 nm), with the optimal size achieved at the 6/1 ratio. The preservation of substantial positive charge suggests superior cellular interaction potential compared with the linear PEG counterpart and challenges the conventional wisdom that all PEG modifications lead to charge shielding.

The control folate-free formulations displayed contrasting assembly characteristics. DSPE-PEG2000/P1500 showed a substantial size increase at the 4/1 ratio (353.3 ± 31.3 nm) with high polydispersity, indicating poor complex stability under low lipid conditions, but achieved a more reasonable size (145.2–215.7 nm) at higher N/P ratios. DSPE-PEG2000/diP1500 maintained more consistent complex sizes across ratios (165.8–214.1 nm) but exhibited a surface charge reduction, particularly at the 6/1 ratio (6.9 ± 0.5 mV). The reference 2X3 system demonstrated highly ratio-dependent complex assembly: at the 4/1 ratio, size increase occurred (318.0 ± 14.5 nm), suggesting suboptimal charge stoichiometry; optimal complex properties were achieved at the 6/1 ratio (155.5 ± 7.3 nm, 18.0 ± 1.4 mV), which corresponds to our previous data [18].

Comparative analysis revealed critical structure–function relationships governing liposomal performance. Liposomal formulations containing the loop-structured PEG lipoconjugate (diP1500) consistently demonstrated superior complex formation characteristics compared with similar formulations with the linear PEG analog, maintaining more favorable surface charges and achieving better size control across N/P ratios. This advantage likely stems from reduced steric hindrance and enhanced flexibility of the loop PEG architecture, representing a significant advancement in PEGylation strategies.

The cytotoxicity of cationic liposomes was evaluated by the MTT assay using KB-3-1 cells. Cells were incubated with PEGylated or conventional liposomes at concentrations ranging from 1–80 μM for 24 h. The IC_50_ values for all tested formulations were not achieved at the maximum concentration of 80 μM. The percentage of viable KB-3-1 cells after incubation with 9 μM (maximum work concentration) and 80 μM liposomes is presented in the Supplementary Materials (Table S1). Importantly, the working concentration of liposomes for the in vitro delivery of nucleic acids did not exceed 9 μM, which was substantially lower than concentrations showing any cytotoxic effects. These results are consistent with our previous data [29,35], showing that incorporation of the 2 mol% folate lipoconjugate or loop-structured PEG derivatives into liposomal formulations does not alter the inherently low toxicity of the base 2X3/DOPE formulation.

The superior performance of diP1500 over P1500 can be rationalized through several complementary mechanisms. Surface packing and architecture: Linear PEG chains (P1500) with single anchor points adopt an extended brush-like conformation at 2 mol%, creating a dense corona that effectively shields the underlying positive charges of 2X3, explaining the dramatic zeta potential reduction for F13/P1500 complexes (3.9–9.1 mV). In contrast, loop-structured PEG with two anchor points (diP1500) forms surface-attached loops, creating a fundamentally different architecture with more efficient surface coverage per molecule, lower effective layer height, a porous corona permitting better charge exposure, and reduced steric hindrance for cellular interactions. This explains why diP1500 maintained a substantial positive charge (21.6–30.5 mV) despite a similar molecular weight.

Protein resistance and anchoring stability: The enhanced circulation of DSPE-PEG2000/diP1500 versus DSPE-PEG2000/P1500 at early time points suggests superior opsonization resistance, possibly through higher local PEG density at the membrane–water interface and more flexible response to approaching proteins. Additionally, loop-structured conjugates with two anchor points exhibited significantly enhanced membrane retention compared with single-anchored linear PEG as simultaneous desorption of both anchors is far less probable, contributing to sustained circulation protection.

Dynamic behavior and siRNA interaction: The constraint of both termini anchored to the surface may allow for dynamic breathing motions, creating transient accessibility to the cationic surface. The preserved positive charge of F13/diP1500 complexes suggests that loop-structured PEG interferes less with siRNA binding, reflecting reduced steric exclusion and allowing more complete complexation while maintaining the protective PEG layer. These mechanisms likely act synergistically to produce the observed superior performance.

The incorporation of folate showed minimal impact on the fundamental physicochemical properties, with folate-containing and folate-free formulations exhibiting comparable size and charge characteristics. This suggests that folate targeting effects are primarily mediated through receptor interactions rather than bulk physicochemical modifications [38]. The N/P ratio emerged as the critical parameter governing complex stability and properties. All formulations showed a ratio-dependent behavior, but optimal performance windows varied significantly. F13 and F13/diP1500 maintained reliable properties across all ratios, while other formulations exhibited more pronounced ratio sensitivity, particularly at extreme N/P values, which is consistent with the published data [37].

### 2.2. Accumulation of siRNA/Liposome Complexes in KB-3-1 Cells

**Figure 2:**
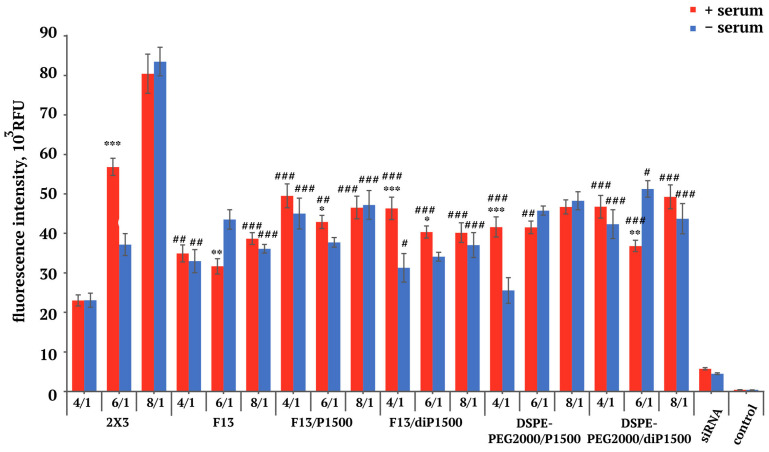
Accumulation of siRNA/liposome complexes in KB-3-1 cells in folate-free conditions (RPMI medium, +/− 5% FBS), 4 h after transfection. * *p* ≤ 0.05, ** *p* ≤ 0.01, *** *p* ≤ 0.001 compared with the serum-free condition, # *p* ≤ 0.05, ## *p* ≤ 0.01, ### *p* ≤ 0.001 compared with 2X3 at the same N/P ratio.

In the first stage, we studied the accumulation of fluorescently labeled siRNA in KB-3-1 cells cultured in folate-deficient medium in the presence of serum and in the serum-free condition. 2X3 demonstrated pronounced N/P ratio dependence with distinct serum response patterns (Figure 2). At the 4/1 N/P ratio, these liposomes exhibited the poorest performance among the tested formulations (approximately 23,000 RFU), with nearly identical efficiency in both the serum and serum-free conditions, indicating inadequate complex stability at low lipid concentrations. The transition to the 6/1 ratio revealed improvement in 2X3 performance, with particularly enhanced efficiency in serum-containing (56.8 × 10^3^ RFU) compared with the serum-free conditions (37.1 × 10^3^ RFU). This unexpected serum enhancement suggests that interactions with plasma protein may facilitate lipoplex stability or promote alternative cellular uptake pathways at this specific charge ratio, which has been reported for certain cationic formulations and may result from protein corona formation [39,40,41].

Optimal transfection efficiency for 2X3 was achieved at the 8/1 ratio, reaching maximum fluorescence values of 80,438–83,493 RFU with minimal difference between the serum +/− conditions. These efficiency levels were comparable to or exceeded those reported for commercial transfection reagents in similar cell lines, confirming the potency of the 2X3 system [29].

Folate-targeted F13 liposomes exhibited remarkably consistent transfection profiles across all tested N/P ratios, maintaining fluorescence levels within the 31–43 × 10^3^ RFU range. This stability suggests that the folate-PEG conjugate provides inherent lipoplex stabilization that compensates for suboptimal N/P ratios. At the 6/1 ratio, F13 demonstrated superior performance in serum-free conditions (43.5 × 10^3^ RFU) relative to the serum-containing medium (31.6 × 10^3^ RFU), sharply contrasting with the 2X3 behavior and indicating distinct cellular uptake mechanisms. Despite consistent performance, the F13 formulations never achieved the peak efficiency observed with 2X3 at high N/P ratios, suggesting a trade-off between targeting specificity and maximum transfection efficiency, similar to what was discussed in Reddy et al. [42].

PEGylated formulations F13/P1500 and F13/diP1500 showed transfection efficiencies comparable to F13 alone, ranging from 37–49 × 10^3^ RFU across all experimental conditions. Formulation with the linear PEG-1500 shield (F13/P1500) demonstrated reduced serum sensitivity with nearly equal performance in both media conditions across all N/P ratios, suggesting effective protection from serum protein interactions [21]. The loop-structured PEG formulation (F13/diP1500) exhibited a serum enhancement pattern at the 4/1 ratio (46.3 × 10^3^ vs. 31.2 × 10^3^ RFU), mirroring the behavior of 2X3 observed at higher N/P ratios.

Comparison between the folate-containing and folate-free formulations revealed no consistent advantage for folate targeting in this in vitro experimental model. DSPE-PEG2000/P1500 and DSPE-PEG2000/diP1500 controls demonstrated transfection efficiencies generally similar to their folate-containing counterparts across most conditions (25–51 × 10^3^ RFU range). This finding challenges the assumption that folate receptor targeting provides significant enhancement for in vitro transfection in KB-3-1 cells, possibly due to competing uptake mechanisms or limitations in the experimental timeframe [43,44].

**Figure 3:**
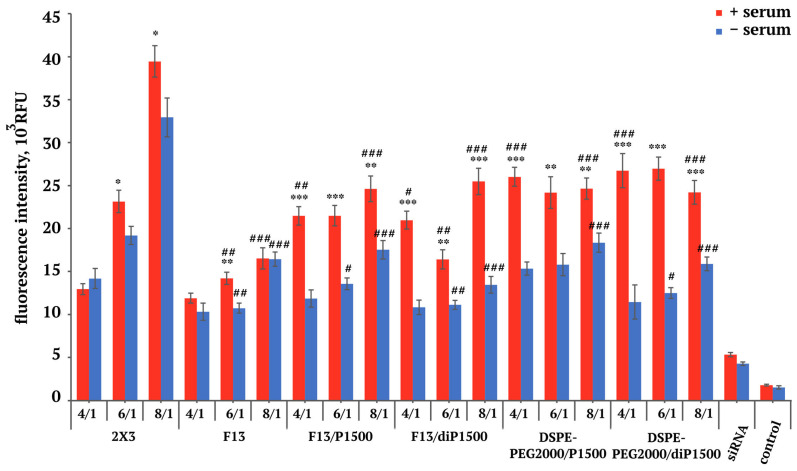
Accumulation of siRNA/liposome complexes in KB-3-1 cells in folate-containing conditions (DMEM medium, +/− 5% FBS), 4 h after transfection. * *p* ≤ 0.05, ** *p* ≤ 0.01, *** *p* ≤ 0.001 compared to -serum condition, # *p* ≤ 0.05, ## *p* ≤ 0.01, ### *p* ≤ 0.001 compared with 2X3 at the same N/P ratio.

At the next stage, we determined the accumulation of the same siRNA in a complex with the studied liposomes in a DMEM medium containing physiological concentrations of folate, under conditions of +\− serum. The addition of free folate to the culture medium significantly altered the transfection performance of all tested formulations (Figure 3). The presence of free folic acid in the growth medium can lead to its interaction with cationic liposomes, changing their charge, size, and delivery efficiency, as we have shown previously [30]. The reference 2X3 maintained its characteristic N/P ratio dependence but with substantially reduced overall efficiency, ranging from 12.9–39.4 × 10^3^ RFU compared with the 23–83 × 10^3^ RFU range observed in folate-free conditions. Notably, the pronounced serum enhancement previously observed at the 6/1 ratio was completely abolished in the presence of folate. This alteration suggests that folate in the medium fundamentally alters the cellular endocytosis pathways or competes with liposomal uptake mechanisms [45]. It should also be taken into account that the compositions of the RPMI media without folate and complete DMEM differed not only in the folate content, but also in the composition of the medium itself, which can also affect the efficiency of transfection.

Folate-containing F13 liposomes experienced the most significant reduction in transfection efficiency, dropping down to the 10,313–16,527 RFU range in folate-containing medium. This substantial decrease provides direct evidence that F13 uptake is folate receptor-mediated, as free folate in the medium directly competes for receptor binding sites, serving as definitive proof of receptor-mediated uptake [42,44].

PEGylated formulations F13/P1500 and F13/diP1500 demonstrated enhanced relative performance in the folate-containing medium compared with F13 alone, particularly at higher N/P ratios. F13/P1500 showed improved efficiency in serum-containing conditions across all ratios, with the most pronounced difference at N/P 4/1 (21,476 vs. 11,860 RFU). This serum-mediated enhancement pattern demonstrates that PEG shields may interact favorably with serum components in a folate-containing environment, possibly through protein corona formation, which facilitates alternative uptake pathways [46].

Remarkably, the folate-free control formulations DSPE-PEG2000/P1500 and DSPE-PEG2000/diP1500 outperformed their folate-containing counterparts in folate-rich medium, particularly in serum-containing conditions. DSPE-PEG2000/P1500 provided 24,000–26,000 RFU at lower N/P ratios, while DSPE-PEG2000/diP1500 maintained similar performance levels. This unexpected superiority also indicates that when folate receptors are saturated by free folate, non-specific uptake mechanisms become more efficient than competitive receptor-mediated endocytosis.

To assess the functional activity of the delivered siRNA, we evaluated the silencing of MDR1 gene expression using KB-3-1-MDR-GFP cells, which stably express the chimeric MDR1-GFP fusion protein equipped with a rapid degradation signal (Table S2, Supplementary Materials). All tested formulations demonstrated gene silencing activity compared with the untreated control cells. The standard 2X3/DOPE formulation reduced the MDR1-GFP expression to 54.5 ± 0.5% of the control levels. Formulations containing folate and protective modifications also demonstrated silencing efficacy. These results demonstrate that the inclusion of PEG-lipoconjugates, particularly variants with a loop structure, does not impair siRNA functionality while providing improved physicochemical properties required for in vivo use. Thus, efficient cellular uptake translates into functional biological activity. Importantly, the maintained inhibitory efficacy of PEGylated formulations confirms their potential for systemic therapeutic use where both stability and biological activity are required.

Thus, comprehensive analysis revealed several critical insights for liposomal delivery system optimization. The N/P ratio emerged as the primary determinant of transfection efficiency, with different formulations requiring distinct ratios for optimal performance. Serum effects were highly formulation-specific and N/P ratio-dependent, with some combinations showing enhancement while others demonstrated inhibition [47]. The absence of consistent folate targeting advantage in standard conditions, combined with the dramatic reduction in folate-targeted efficiency at physiological folate concentrations, highlights the importance of evaluating delivery systems under conditions that accurately reflect the target environment. These findings provide crucial guidance for therapeutic applications: 2X3 at high N/P ratios offers superior in vitro performance for research applications, while PEGylated systems provide more consistent performance across varying physiological conditions.

### 2.3. siRNA Concentration Dynamics in Mouse Blood Plasma

**Figure 4:**
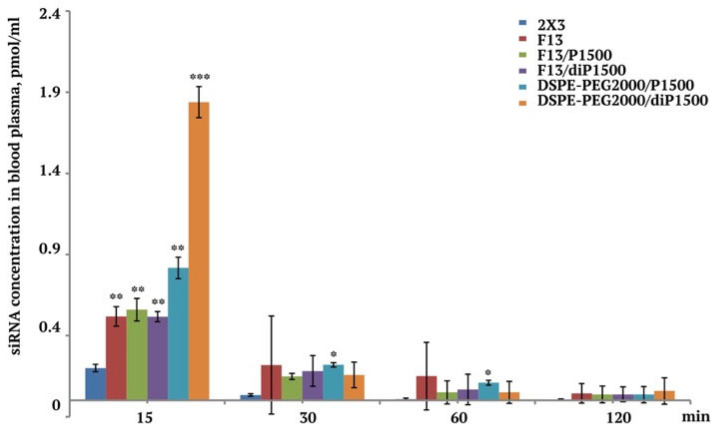
siRNA circulation dynamics in mouse blood plasma after IV injection of lipoplexes (N/P 4/1). * *p* ≤ 0.05, ** *p* ≤ 0.01, *** *p* ≤ 0.001 compared with 2X3 at the same time point.

The duration of drug circulation in the bloodstream is important to ensure its accumulation in the organs; therefore, we studied the dynamics of siRNA concentration after its intravenous administration pre-complexed with the studied liposomes to CBA mice. The evaluation of siRNA concentration dynamics in mouse blood plasma revealed distinct pharmacokinetic profiles among the tested liposomes, though statistical significance as limited to specific time points and liposome formulations (Figure 4). Quantitative analysis using stem-loop PCR demonstrated that even though numerical differences existed throughout the observation period, statistically significant variations were confined to the early distribution phase. This limited statistical significance at later time points is common in pharmacokinetic studies with small sample sizes and highlights the challenges in achieving adequate statistical power for circulation studies.

At 15 min post-injection, clear statistical differences emerged among the compounds. DSPE-PEG2000/diP1500 demonstrated a significantly higher plasma concentration (1.84 ± 0.09 pmol/mL) compared with all other tested liposomes (*p* < 0.001), establishing this loop-structured PEG control as the most effective system for immediate post-injection circulation. This superior initial circulation of the loop-structured PEG formulation supports our in vitro findings and suggests that loop PEG architecture may provide enhanced protection from immediate clearance mechanisms, similar to that described by Abu Lila et al. [48]. DSPE-PEG2000/P1500 also achieved significantly elevated initial plasma levels (0.82 ± 0.07 pmol/mL) relative to other formulations (*p* < 0.05), though the difference was less pronounced than its loop-structured counterpart. The superior performance of both PEGylated control formulations confirms the well-established benefits of PEGylation for circulation enhancement [21]. The reference 2X3 system showed significantly lower initial concentrations (0.20 ± 0.02 pmol/mL) compared with the folate-targeted PEGylated formulations F13/P1500 and F13/diP1500 (*p* < 0.01), confirming the substantial circulation advantage provided by PEG modification. This difference in initial circulation between PEGylated and non-PEGylated formulations is consistent with classical established principles of stealth liposome design [49]. Folate-targeted systems F13 (0.52 ± 0.06 pmol/mL), F13/P1500 (0.56 ± 0.07 pmol/mL), and F13/diP1500 (0.52 ± 0.03 pmol/mL) achieved comparable initial plasma levels without significant differences among themselves, suggesting that folate conjugation via the PEG2000 linker (2%) provides consistent circulation benefits regardless of the additional PEG component (up to 4%).

The difference in blood concentrations 15 min after the administration of lipoplexes with F13/P1500 and DSPE_PEG2000-P1500 was noteworthy, as was an even more significant difference in the concentrations of lipoplexes with F13/diP1500 and DSPE-PEG2000/diP1500, which differed in pairs only by the presence of a folate molecule at the end of the PEG2000 chain. It should be noted that the in vitro experiments did not show such significant differences. It can be assumed that the presence of folate can affect both the hydrophobicity of the particle surface and the interaction with serum proteins, and accordingly, the size and composition of the protein corona [50,51].

At 30 min, DSPE-PEG2000/P1500 showed the highest plasma concentration (0.22 ± 0.01 pmol/mL), followed by F13 (0.22 ± 0.30 pmol/mL), though these differences fell within statistical variability. The maintenance of relatively high concentrations at 30 min for these liposomes suggests sustained circulation benefits that could translate to enhanced therapeutic efficacy.

By 120 min, all formulations approached the baseline concentrations, with F13 showing the highest numerical retention (0.04 ± 0.06 pmol/mL), though again without statistical significance. The siRNA/2X3 formulation reached near-complete elimination (0.01 ± 0.003 pmol/mL), maintaining the circulation disadvantage established at earlier time points. The rapid clearance of non-PEGylated formulations was expected and consistent with established pharmacokinetic principles for cationic lipoplexes [52].

The statistically verified superiority of DSPE-PEG2000/diP1500 over DSPE-PEG2000/P1500 at 15 min suggests that loop-structured PEG modifications may provide enhanced immediate circulation benefits compared with linear PEG architectures. This finding represents the first direct comparison of linear versus loop-structured PEG modifications in vivo and suggests that polymer architecture may be as important as molecular weight for circulation enhancement.

The consistent statistical disadvantage of 2X3 relative to the PEGylated formulations confirms that surface modification is essential for initial circulation protection. The early circulation phase is often most critical for therapeutic efficacy, as this determines the amount of drug that reaches the target tissues before clearance [47]. Therefore, liposomes with low first-pass kidney clearance may provide therapeutic advantages, even if the long-term circulation differences are not statistically significant.

Differences at the 15 min time point established that the PEGylated control formulations, particularly with the loop-structured PEG architecture, provided superior protection from immediate clearance mechanisms, likely through enhanced resistance to protein opsonization and delayed recognition by reticuloendothelial clearance pathways. The mechanism of enhanced circulation likely involves the formation of a hydrophilic PEG corona that prevents protein adsorption and subsequent recognition by macrophages [34]. The superior performance of loop-structured PEG may result from more efficient surface coverage or altered protein binding characteristics compared with linear PEG.

For therapeutic applications, formulation selection should prioritize the statistically verified early phase circulation advantages of DSPE-PEG2000/diP1500 and DSPE-PEG2000/P1500 while acknowledging that longer-term circulation benefits remain to be definitively established through studies with increased statistical power. The observed trends, while suggestive of potential advantages, require validation through additional pharmacokinetic studies with larger sample sizes to achieve statistical significance beyond the initial distribution phase.

The integration of these findings with our in vitro results revealed important structure–function relationships that support the superiority of loop-structured PEG modifications. The enhanced circulation of DSPE-PEG2000/diP1500 correlates with its good in vitro stability and complex formation properties, suggesting that the benefits of loop PEG architecture extend across multiple performance parameters. This consistency across different evaluation criteria strengthens the case for the further development of loop-structured PEG modifications for siRNA delivery applications.

DSPE-PEG2000/diP1500 achieved 1.84 ± 0.01 pmol/mL at 15 min (improvement over non-PEGylated 2X3), consistent with the literature for 2–5-fold improvements for PEG2000-liposomes [21], with a statistically significant advantage over the linear PEG of identical molecular weight—a comparison not previously demonstrated. For charge preservation, F13/diP1500 maintained 21.6–30.5 mV versus F13/P1500’s 3.9–9.1 mV. The literature reports a 50–80% charge reduction for PEG-modified cationic liposomes [6,53], while our loop-structured approach preserved 65–85% of positive charge.

The PEG dilemma—enhanced circulation but impaired uptake—has prompted several strategies: cleavable PEG requiring precise kinetics tuning [24], low-density PEGylation (1–2 mol%) [20], short PEG chains with less circulation benefit, and mixed PEG populations adding complexity. Our loop-structured PEG achieved a 2–3× higher surface charge than the linear PEG while maintaining equivalent circulation without cleavable bonds or mixed populations. This challenges the assumption that PEG-mediated stabilization inevitably causes charge shielding, providing a design principle for diverse PEGylated nanocarriers including LNPs and polymeric systems.

The demonstrated advantages may extend to mRNA (enhanced protection without excessive shielding, building on our previous work [19], plasmid DNA (better charge preservation for condensation), antisense oligonucleotides (improved pharmacokinetics), CRISPR/Cas9 (co-delivery with reduced immunogenicity), and immunostimulatory RNA (prolonged circulation for immune activation [29]. Cargo-specific optimization and in vivo validation are required. The loop-structured PEG architecture addresses a cargo-independent challenge, suggesting broad applicability.

Successful siRNA delivery systems require integrated optimization across multiple parameters, including physicochemical properties, cellular uptake, and in vivo performance, rather than focusing on individual characteristics in isolation. Optimal lipid nanoparticle design requires balancing multiple competing factors including stability, immunogenicity, targeting efficiency, and manufacturing considerations based on recent clinical experience.

## 3. Materials and Methods

### 3.1. Lipoconjugate Synthesis and Liposome Preparation

PEG-lipids P1500 and diP1500 were synthesized as described earlier [18,54]. All liposomal formulations were prepared by the hydrating thin lipid film method [55]. A solution of the polycationic lipid 1,26-bis(cholest-5-en-3β-yloxycarbonylamino)-7,11,16,20-tetraazahexacosan tetrahydrochloride (2X3) [56] in a mixture of CHCl_3_/CH_3_OH (5/1, *v*/*v*) was added to a solution of 1,2-dioleoyl-*sn*-glycero-3-phosphoethanolamine (DOPE, Avanti Polar Lipids, Alabaster, AL, USA) in CHCl_3_ at a molar ratio of 1:2 and gently stirred. A solution of PEG-lipids P1500, diP1500, DSPE-PEG2000 (Lipoid, Ludwigshafen am Rhein, Germany), or/and DSPE-PEG2000-folate (Avanti Polar Lipids, Alabaster, AL, USA) (2 mol%) in CHCl_3_/CH_3_OH (1/1, *v*/*v*) was added, and organic solvents were removed in vacuo. The obtained lipid film was dried for 4 h at 0.1 Torr to remove residual organic solvents and was hydrated in water for injection (Solopharm, Moscow, Russia) at 4 °C overnight. The resulting liposomal dispersion was sonicated for 15 min at 70–75 °C in a bath-type sonicator Sonorex Digitec DT 52H (Bandelin electronic, Berlin, Germany), flushed with argon, and stored at 4 °C. In the resulting dispersion, the cationic lipid 2X3 concentration was 1 mM.

Mass spectrometry for P1500 was described early in our article [18] and in patent RF No. 2,683,572. The peak with the maximal intensity of PEG for P1500 (m = 2, n = 34) was chosen to calculate the molecular ion. P1500 (m = 2, n = 20–46): MALDI-MS, *m/z*: [M + H] + calculated for C_106_H_213_N_2_O_39_, 2138.475; found: 2138.272. The synthesis of diP1500 was described previously in our article [57]. The peak with the maximal intensity of PEG for diP1500 (m = 2, n = 39) was chosen to calculate the molecular ion. diP1500 (m = 2, n = 25–43): ESI-MS, *m/z*: [M + K] + calculated for C_146_H_292_KN_2_O_47_, 2865.016; found: 2865.0400.

### 3.2. Synthesis of siRNAs and siRNA/Liposome Complex Preparation

The synthesis of the 2′-O-methylated anti-siMDR1 (sense strand 5′-GGCUUmGACmAAGUUmGUmAUmAUmGG-3′; antisense strand 5′-AUmAUmACmAACUUmGUCmAAGCCmAA-3′) and siScr siRNAs (sense strand 5′-GCUUGAAGUCUUUmAAUUmAAGG-3′; antisense strand 5′-UUmAAUUmAAAGACUUCmAAGCGG-3′) used in this study was carried out as previously described [31]. Cyanine 5.5 (Cy5.5) was attached to siMDR1 through a 3′-aminohexyl linker according to the manufacturer’s protocol, using the Cy5.5 *N*-hydroxysuccinimide ester (Biotech Industry Ltd., Moscow, Russia) in 0.1 M Tris buffer (pH 8.4). Strands of siRNAs (50 μM) were annealed in a buffer containing 30 mM HEPES-KOH (pH 7.4), 100 mM sodium acetate, and 2 mM magnesium acetate by heating at 90 °C for 5 min, followed by cooling to room temperature. The siRNA preparations were stored at −20 °C until use. For the in vivo studies, the cationic liposomes and siRNA complexes (at 4/1 N/P ratio of positively-charged amine (N) groups to negatively-charged nucleic acid phosphate (P)) were formed in a volume of 200 µL by mixing 100 µL siRNA (final concentration 7 µM) and 100 µL liposome at the corresponding concentration. For the in vitro studies, the cationic liposomes and siRNA complexes at different N/P ratios (2/1, 4/1, 8/1) were formed in a volume of 100 µL by mixing 50 µL siRNA (final concentration 5 µM) and 50 µL liposome at the corresponding concentration. All solutions were prepared in serum-free OptiMEM (Invitrogen, Waltham, MA, USA). The resulting mixtures were incubated for 20 min at room temperature before IV administration to mice or before being added to the cells.

### 3.3. Liposome Sizes and Zeta Potentials

Particle size and zeta potential were assessed using a Zetasizer Nano ZS (Malvern Panalytical Ltd., Malvern, UK). The average hydrodynamic diameter was determined from the particle number distributions, and these measurements were repeated three times. For this, 50 µL of siRNA solution prepared in Opti-MEM was mixed with 50 µL of liposome solution in Opti-MEM taken at the appropriate N/P ratio. After incubating at room temperature for 20 min, the size analysis was conducted using a 100 µL microcuvette. Liposomal morphology was additionally characterized by transmission electron microscopy (TEM) to validate the size distributions obtained by DLS and confirm the particle structure. TEM images of all liposome formulations are provided in the Supplementary Materials (Figure S1). To determine the zeta potential, 900 µL of MilliQ water was added to the sample, and the surface potential was recorded in a 1 mL cuvette; these measurements were repeated three times.

All physicochemical characterizations were performed at pH 7.2–7.4, representing the physiological pH during circulation and cellular uptake. This choice reflects the mechanistic basis of our delivery system: the 2X3/DOPE formulations maintained structural stability at physiological pH (7.2–7.4), while endosomal escape was mediated by DOPE-induced membrane fusion triggered by endosomal acidification rather than by pH-dependent destabilization of the carrier itself.

### 3.4. Animals

Adult male CBA mice (weighing 20–24 g) were obtained from the Center for Genetic Resources of Laboratory Animals at the Institute of Cytology and Genetics SB RAS. These animals were housed in the vivarium of the Institute of Chemical Biology and Fundamental Medicine, SB RAS, under natural light conditions and provided with a standard laboratory diet, adhering to international guidelines for the proper use and care of laboratory animals (ECC Directive 86/609/EEC). The experimental protocol was approved by the Committee on the Ethics of Animal Experiments at the Institute of Cytology and Genetics of SB RAS (protocols #22.11 from 30 May 2014 and #220 from 6 March 2025).

### 3.5. Tumor Cell Line

Human cervical carcinoma KB-3-1 cells were obtained from the Russian Cell Culture Collection at the Institute of Cytology, Russian Academy of Sciences, St. Petersburg, Russia. These cells were cultured in DMEM (Sigma-Aldrich, St. Louis, MO, USA) supplemented with 10% FBS (HyClone, GE Healthcare, Chicago, IL, USA) and 1% antibiotic-antimycotic solution (MP Biomedicals, Santa Ana, CA, USA). Cells were grown in a humidified environment with 5% CO_2_ and 95% air at 37 °C and were regularly passaged to maintain exponential growth.

### 3.6. Cellular Accumulation Assay

One day prior to the experiment, KB-3-1 cells in the exponential growth phase were plated onto 48-well plates at a density of 5.5 × 10^4^ cells per well. For serum-containing conditions, Cy5.5-labeled siRNA or its complexes with liposomes were added to the cells 24 h after plating. For serum-free conditions, the complete medium was changed to serum-free medium before the addition of lipoplexes. For folate-free conditions, folate-depleted RPMI 1640 medium with 5% FBS was used instead of DMEM (Thermo Fisher Scientific, Waltham, MA, USA). Four hours after the addition of Cy5.5-siRNA, cells were detached using TrypLE (Thermo Fisher Scientific, Waltham, MA, USA) and fixed in 2% formaldehyde in phosphate-buffered saline (PBS). Cell analysis was conducted using a NovoCyte flow cytometer (ACEA Biosciences, Santa Clara, CA, USA); 15,000 cells from each sample were analyzed.

### 3.7. Quantitative Stem-Loop Real-Time PCR Analysis of the Dynamics of siRNA Concentration in Blood Plasma

For plasma preparation, 50 µL blood samples were collected from the retro-orbital sinus of the CBA mice 15, 60, and 120 min after the IV injection of 0.5 µg/g siMDR/liposome complexes (N/P 4/1). The RNA from plasma samples was isolated by heating in Triton [58]. All siRNA specific stem-loop primers for RT-qPCR analysis were designed according to the instructions of Czimmerer [59].

The reverse transcription reaction was performed in a final volume of 40 µL containing 4 µL of Triton X-100 preheated plasma supernatant, 8 µL 10 µM siMDR1 specific stem loop-RT primer (5′-GTTGGCTCTGGTGCAGGGTCCGAGGTATTCGCACCAGAGCCAACCATCAG-3′), 8 µL sterile water, and 20 µL of Master Mix prepared from a M-MuLV–RH Reverse Transcription Kit (Biolabmix, Novosibirsk, Russia). The reaction mixture was incubated at 42 °C for 1 h. The cDNA was amplified in a total volume of 50 µL containing 10 µL of cDNA template, 10 µL siMDR specific forward (5′-GTTGGGGATATACAACTTGTCA-3′) and reverse (5′-GTGCAGGGTCCGAGGT-3′) primers (1 µM), 5 µL sterile water, and 25 µL of BioMaster HS-qPCR SYBR Blue master mix with SYBR Green I fluorescent dye (Biolabmix, Novosibirsk, Russia) using an CFX96 real-time system (Bio-Rad, Hercules, CA, USA) according to the following scheme: one cycle—5 min, 95 °C; 35 cycles—10 s, 95 °C; 30 s, 51 °C; and 30 s, 72 °C. All measurements were conducted in triplicate.

### 3.8. Statistical Analysis

Statistical analysis was conducted using GraphPad Prism 8.4.3 software (GraphPad Software, Inc., San Diego, CA, USA), and the data are presented as the mean ± standard deviation (SD). Statistically significant differences were determined using an ordinary two-way ANOVA with Dunnett’s multiple comparisons test. The differences between the values were considered statistically significant when *p* < 0.05.

## 4. Conclusions

This study demonstrated the fundamental advantages of loop-structured PEG architectures for siRNA delivery, revealing consistent improvements in physicochemical properties, complex formation, and initial in vivo circulation. The F13/diP1500 formulation emerged as the most promising targeted system, combining folate receptor specificity with favorable complex properties across multiple N/P ratios. The discovery that loop PEG structures outperformed linear analogs represents an advancement in nanoparticle design with potential applications beyond siRNA delivery.

The critical role of N/P ratio as the primary determinant of efficiency emphasizes the necessity for formulation-specific optimization, while the complex interactions between formulation composition, serum components, and physiological ligand concentrations underscore the importance of physiologically relevant testing conditions. The competitive inhibition of folate-targeted uptake by free folate provides definitive proof of the receptor-mediated mechanism, validating the targeting strategy. The statistically significant circulation advantages of loop-structured PEG formulations in the critical early phase, combined with their superior in vitro characteristics, support their potential for clinical translation.

Several limitations of this study should be acknowledged. The biodistribution studies employed limited sample sizes (n = 3 mice per group), and therapeutic efficacy in tumor models was not evaluated. While our work provides strong evidence for loop-structured PEG superiority, several questions merit further investigation: optimal loop size (PEG1500 tested; PEG1000–2000 comparison needed); anchor group spacing effects; performance under pathological conditions (inflammation, altered glycocalyx); long-term circulation beyond early time points; and molecular dynamics simulations for mechanistic elucidation. Additionally, we only investigated PEG1500 molecular weight and folate targeting; systematic evaluation of other PEG lengths and targeting ligands would allow for more general conclusions to be drawn. Future studies should prioritize tumor efficacy evaluation, comprehensive immunogenicity assessment including anti-PEG antibodies and accelerated blood clearance phenomena [48,60], scale-up for clinical manufacturing, and extension to alternative nucleic acid cargos such as mRNA and CRISPR components.

Despite these limitations, the consistent advantages of loop-structured PEG across multiple evaluation criteria establish a strong foundation for translational development. Further elucidation of the molecular mechanisms underlying PEG loop structure efficacy and exploration of their applicability to other target ligands and therapeutic cargoes will maximize the therapeutic potential of this innovative nanoparticle design approach.

## Figures and Tables

**Table 1:** The composition and characteristics of the cationic liposomes used in the study and their complexes formed with siRNA.

Liposome	Target Component	PEG Component		Z-Ave, d.nm	PdI	ZP, mV
**F13**	DSPE-PEG2000-folate 2%	-		128.6 ± 5.9	0.7	32.4 ± 9.0
			4/1	156.7 ± 12.3	0.5	23.1 ± 3.8
			6/1	144.6 ± 22.4	0.6	30.5 ± 5.1
			8/1	158.2 ± 10.2	0.4	35.4 ± 2.4
**F13/P1500**	DSPE-PEG2000-folate 2%	P1500 2%		241.8 ± 65.7	0.4	39.2 ± 8.1
			4/1	201.5 ± 39.4	0.4	3.9 ± 3.3
			6/1	191.1 ± 5.5	0.5	7.4 ± 1.2
			8/1	171.7 ± 8.6	0.5	9.1 ± 0.3
**F13/diP1500**	DSPE-PEG2000-folate 2%	diP1500 2%		126.0 ± 23.0	0.4	28.8 ± 12.8
			4/1	214.7 ± 14.0	0.5	25.4 ± 3.5
			6/1	138.9 ± 8.3	0.7	30.5 ± 7.3
			8/1	172.5 ± 113.6	0.8	21.6 ± 7.6
**DSPE-PEG2000/P1500**	-	DSPE-PEG2000 2% P1500 2%		116.1 ± 17.9	0.7	26.8 ± 4.7
			4/1	353.3 ± 31.3	0.5	36.6 ± 13.5
			6/1	215.7 ± 13.7	0.4	25.0 ± 7.5
			8/1	145.2 ± 44.9	0.7	35.6 ± 10.1
**DSPE-PEG2000/diP1500**	-	DSPE-PEG2000 2% diP1500 2%		198.0 ± 99.2	0.5	26.8 ± 2.0
			4/1	165.8 ± 21.6	0.3	14.0 ± 3.6
			6/1	202.3 ± 22.7	0.5	6.9 ± 0.5
			8/1	214.1 ± 38.4	0.5	35.5 ± 2.6
**2X3**	-	-		88.0 ± 7.1	0.2	39.5 ± 2.2
			4/1	318.0 ± 14.5	0.4	21.6 ± 1.6
			6/1	155.5 ± 7.3	0.3	18.0 ± 1.4
			8/1	238.5 ± 24.1	0.4	23.8 ± 0.2

## Data Availability

The raw data supporting the conclusions of this article will be made available by the authors on request.

## Supplementary Materials

The following supporting information can be downloaded at: https://www.mdpi.com/article/10.3390/molecules30204127/s1. Table S1. Cytotoxicity assessment of liposomal formulations. The percentage of viable KB-3-1 cells after 24 h incubation with 9 or 80 μM liposomes, determined by MTT assay. Data are presented as mean ± SD (n = 3). Table S2. Silencing activity of siMDR delivered by cationic liposomes in KB-3-1-MDR-GFP cells. The cells were incubated with the lipoplexes formed by cationic liposomes at 8/1 N/P ratios. The levels of MDR1-GFP expression in the cells were evaluated using flow cytometry after 72 h of incubation with the lipoplexes. Levels of MDR1-GFP expression in the control cells (without any treatment) were set at 100%. The differences between mean values for 2X3/DOPE and other preparations at the same N/P were considered statistically significant: *** *p* < 0.01, *** *p* < 0.001 (Mann-Whitney U-test). Figure S1. TEM visualization of liposomes was performed using the negative contrast method. A 10 µL drop of sample (100-fold dilution of 1 mM stock solution) was adsorbed for 1 min on the copper grid covered with formvar film and excess liquid was pulled back by a pipette. Then a grid was placed for 10 s on a drop of 0.5% uranyl acetate, excess liquid was removed with filter paper and grids were air dried. The samples were studied on a JEM 1400 TEM (Jeol, Japan) equipped with a Veleta digital camera (EM SIS, Germany).
