# Supplementary material for: Loop-Structured PEG-Lipoconjugate Enhances siRNA Delivery Mediated by Liner-PEG Containing Liposomes
Source: Molecules. 2025 Oct 19;30(20):4127. doi: 10.3390/molecules30204127 (PMC12566006; doi:10.3390/molecules30204127)
Supplement: Supplementary file 1 [file molecules-30-04127-s001.zip › Supplementary.pdf]

Cationic Liposome	Living Cells, %	
	9 μM liposome	80 μM liposome
F13	98.7 ± 0.2	65.3 ± 1.1
F13/P1500	99.1 ± 0.6	64.2 ± 2.7
F13/diP1500	99.4 ± 0.6	60.7 ± 3.9
DSPE-PEG2000/P1500	98.9 ± 0.2	65.9 ± 6.4
DSPE-PEG2000/diP1500	99.2 ± 0.4	69.7 ± 9.3
2X3	99.2 ± 0.5	71.3 ± 2.5

Table S1. Cytotoxicity assessment of liposomal formulations. The percentage of viable KB-3-1 cells after 24 h incubation with 9 or 80 μM liposomes, determined by MTT assay. Data are presented as mean ± SD (n=3).

Liposome	MDR1-GFP expression, %
F13	85.7 ± 0.7**
F13/P1500	76.1 ± 0.6***
F13/diP1500	78.4 ± 0.6***
DSPE-PEG2000/P1500	77.9 ± 0.2***
DSPE-PEG2000/diP1500	76.2 ± 0.4***
2X3	54.5 ± 0.5

Table S2. Silencing activity of siMDR delivered by cationic liposomes in KB-3-1-MDR-GFP cells. The cells were incubated with the lipoplexes formed by cationic liposomes at 8/1 N/P ratios. The levels of MDR1-GFP expression in the cells were evaluated using flow cytometry after 72 h of incubation with the lipoplexes. Levels of MDR1-GFP expression in the control cells (without any treatment) were set at 100%. The differences between mean values for 2X3/DOPE and other preparations at the same N/P were considered statistically significant: ***$p < 0.01$, ***$p < 0.001$ (Mann-Whitney U-test).

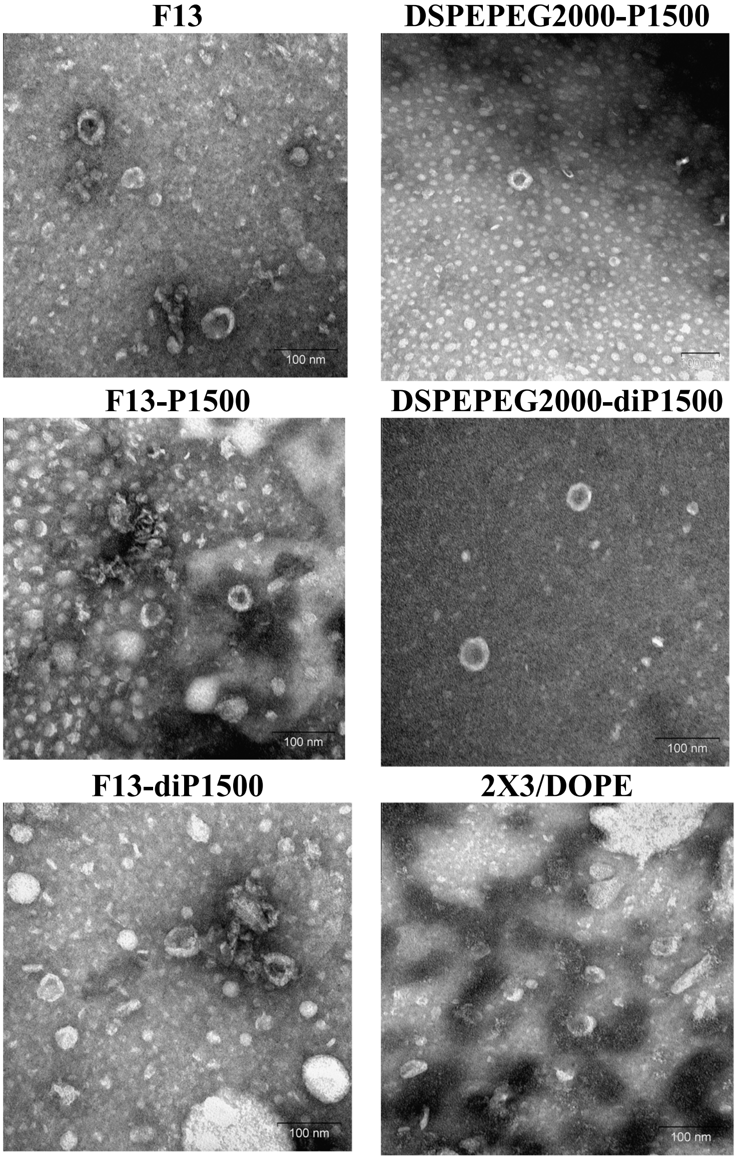

Figure S1. TEM visualization of liposomes was performed using the negative contrast method. A 10 μL drop of sample (100-fold dilution of 1 mM stock solution) was adsorbed for 1 min on the copper grid covered with formvar film and excess liquid was pulled back by a pipette. Then a grid was placed for 10 s on a drop of 0,5% uranyl acetate, excess liquid was removed with filter paper and grids were air dried. The samples were studied on a JEM 1400 TEM (Jeol, Japan) equipped with a Veleta digital camera (EM SIS, Germany).
